# Supplementary material for: One-Step Preparation of PVDF/GO Electrospun Nanofibrous Membrane for High-Efficient Adsorption of Cr(VI)
Source: Nanomaterials (Basel). 2022 Sep 8;12(18):3115. doi: 10.3390/nano12183115 (PMC9503595; doi:10.3390/nano12183115)
Supplement: Supplementary file 1 [file nanomaterials-12-03115-s001.zip › nanomaterials-1895878-supplementary.pdf]

Supporting Information

One-Step Preparation of PVDF/GO Electrospun Nanofibrous Membrane for High-Efficient Adsorption of Cr(VI)

Qingfeng Wang [1,2], Zungui Shao [1,2], Jiaxin Jiang [3], Yifang Liu [1,2], Xiang Wang [3], Wenwang Li[3] and Gaofeng Zheng [1,2,*]

[1] Department of Instrumental and Electrical Engineering, Xiamen University, Xiamen 361102, China
[2] Shenzhen Research Institute of Xiamen University, Shenzhen 518000, China
[3] School of Mechanical and Automotive Engineering, Xiamen University of Technology, Xiamen 361024, China

* Correspondence: zheng_gf@xmu.edu.cn

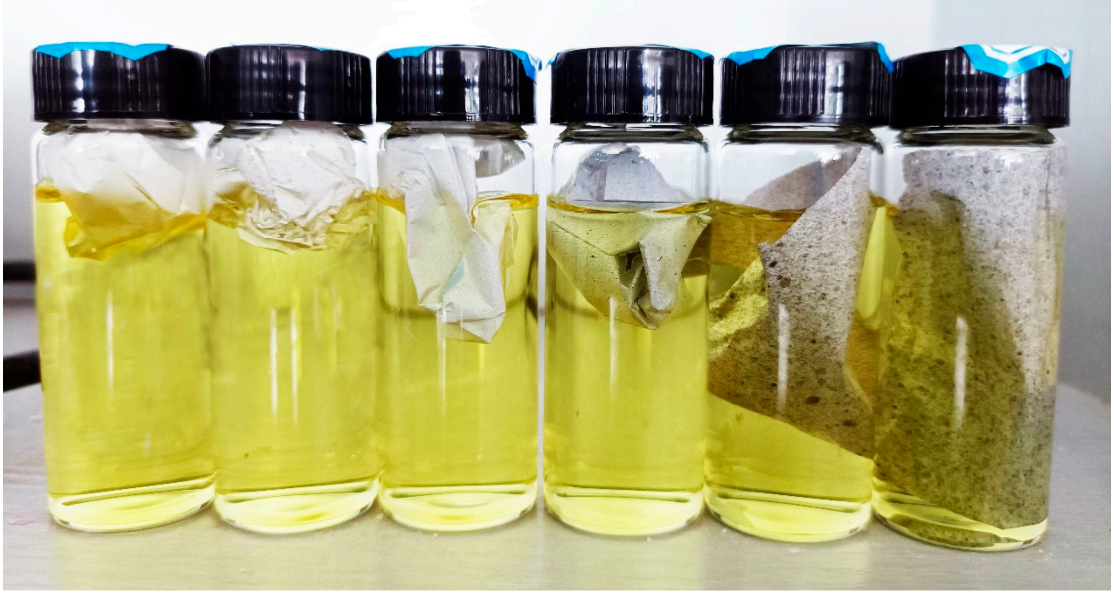

Figure S1. immersion of different nanofibers (left to right: PVDF; PVDF–GO; PVDF@GO; PVDF–2GO; PVDF–1GO@GO; PVDF–2GO@GO).
